# Emergency Department Predictors of Mechanical Ventilation in Pediatric Spine Fracture Patients in the US

**DOI:** 10.1177/21925682261483531

**Published:** 2026-08-24

**Authors:** Samer G. Salman, Rohan A. Phadke, Margaret A. Baldwin

**Affiliations:** 1 School of Medicine, 3989 Baylor College of Medicine, Houston, TX, United States; 2 Joseph Barnhart Department of Orthopedic Surgery, 3989 Baylor College of Medicine, Houston, TX, United States; 3 Orthopedics & Sports Medicine, Texas Children’s Hospital, Houston, TX, United States; 4 Scoliosis and Spinal Deformity Program at Texas Children’s Hospital, Houston, TX, United States

**Keywords:** pediatric, spine fracture, mechanical ventilation, hemodynamic instability, trauma, outcomes, risk factors, emergency department

## Abstract

**Study Design:**

Retrospective cohort study.

**Objectives:**

To identify emergency department variables associated with mechanical ventilation among pediatric patients with spine fractures and characterize outcomes by ventilation duration.

**Methods:**

We queried the National Trauma Data Bank from 2017 to 2022 for patients younger than 18 years with cervical, thoracic, or lumbar vertebral fractures. The primary outcome was mechanical ventilation during hospitalization. Thirty-five ED-available predictors were entered into multivariable logistic regression. Discrimination was assessed using the C-statistic, and robustness was tested across 14 prespecified sensitivity and subgroup models. Secondary outcomes were compared across no ventilation, 1 ventilator day, and 2 or more ventilator days.

**Results:**

Among 29,024 patients, 5,831 (20.1%) received mechanical ventilation. Severe neurologic impairment was the dominant predictor: GCS ≤8 had an adjusted odds ratio (aOR) of 190.52, and GCS 9-12 had an aOR of 15.18. Other independent risk factors included hypotension, penetrating trauma, hypothermia, hypoxemia, shock index ≥0.9, and air transport. Thoracic and lumbar fractures, private vehicle arrival, female sex, interfacility transfer, and helmet use were associated with lower odds. Model discrimination was excellent (C-statistic, 0.928), with consistent performance across sensitivity analyses (C-statistics, 0.916-0.940). Prolonged ventilation occurred in 5,081 patients (17.5%) and was associated with 15.7% mortality, 99.0% ICU admission, median hospital length of stay of 15 days, and higher complication rates.

**Conclusions:**

ED GCS, physiologic instability, respiratory compromise, and cervical fracture location identified pediatric spine fracture patients at higher risk for mechanical ventilation. Early recognition may support triage, airway planning, ICU allocation, and family counseling.

## Introduction

Pediatric spine fractures are increasingly common and can carry substantial morbidity, particularly when accompanied by neurological injury or respiratory compromise.^1^ Among children with vertebral fractures, the need for mechanical ventilation identifies a high-acuity subgroup with greater resource use and worse short- and long-term outcomes. Despite its clinical importance, factors associated with mechanical ventilation in pediatric spine fracture patients are not well defined.

The pediatric spine differs from the adult spine in ways that affect both injury pattern and outcome.^2,3^ Ligamentous laxity, incomplete ossification, and a relatively large head make the immature spine more flexible but also more susceptible to upper cervical injury and spinal cord injury without radiographic abnormality.^2-6^ Because the fulcrum of cervical motion lies at C2-C3 in younger children rather than C5-C6, injury patterns differ from those seen in adults, with younger patients sustaining more upper cervical injuries and experiencing greater mortality when neurological injury occurs.^1,3,4,7^ These differences suggest that predictors derived from adult trauma cohorts may not apply well to children with spine fractures.

Mechanical ventilation in trauma is associated with higher mortality, longer intensive care unit stay, and complications such as ventilator-associated pneumonia, acute respiratory distress syndrome, sepsis, and delirium.^8-11^ In pediatric trauma, ventilation is common among critically injured patients and is associated with worse outcomes.^12^ Prior studies in pediatric cervical spinal cord injury also show age-related differences in ventilator need and duration.^7,13^ Respiratory failure after trauma may reflect pulmonary injury, neurologic compromise, hemodynamic instability, or concomitant traumatic brain injury.^13,14^ However, existing pediatric spine trauma literature has focused mainly on epidemiology and mortality, with mechanical ventilation usually reported as a secondary outcome rather than a primary endpoint for prediction.^1,4,5,7^

Early identification of children with spine fractures who are likely to require mechanical ventilation could improve airway planning, triage, transfer decisions, ICU resource allocation, and family counseling.^15,16^ We aimed to identify emergency department-available variables independently associated with mechanical ventilation in pediatric patients with spine fractures using a national database and to describe the burden of ventilation duration across injury patterns and age groups.

## Methods

### Study Design and Ethics

We performed a retrospective cohort study using National Trauma Data Bank (NTDB) data from 2017 through 2022.^17^ We included pediatric patients with vertebral fractures and estimated adjusted odds ratios (aORs) with 95% confidence intervals. We followed the Strengthening the Reporting of Observational Studies in Epidemiology (STROBE) statement for cohort studies. The Institutional Review Board determined that this deidentified NTDB study did not constitute human subjects research; therefore, formal IRB review was not required.

We identified patients younger than 18 years with vertebral fractures using International Classification of Diseases, Tenth Revision (ICD-10) diagnosis codes for cervical fracture (S12.xxx), thoracic fracture (S22.0xx), and lumbar fracture (S32.0xx). We excluded patients with missing discharge disposition and nonstandard discharge destinations, including discharge against medical advice, police custody, interhospital transfer listed as discharge, hospice, psychiatric facility, and other nonstandard destinations.

### Outcome Definition and Predictor Variables

The primary outcome was mechanical ventilation during hospitalization. For sensitivity analysis, we defined prolonged mechanical ventilation as total ventilator days of 2 or greater to reduce misclassification from brief peri-procedural ventilation.

We analyzed 35 binary predictors available during the initial emergency department evaluation across 13 domains: age group, sex, race/ethnicity, vital signs, body composition, mechanism of injury, trauma type, transport mode, insurance status, substance use, interfacility transfer, fracture level, and protective devices. When continuous data were missing, we coded the corresponding binary abnormality variable as 0 and tested that assumption in sensitivity analyses. We excluded Injury Severity Score because it is not available at bedside.

### Analysis

We compared baseline characteristics between ventilated and nonventilated patients using standardized mean differences (SMDs), with an absolute SMD greater than 0.1 indicating meaningful imbalance. We entered all 35 predictors simultaneously into a multivariable logistic regression model using maximum likelihood estimation. We derived aORs and 95% confidence intervals from Wald statistics and prespecified clinical significance as aOR greater than 1.5 for risk and aOR less than 0.67 for protection. We assessed multicollinearity with variance inflation factors using a threshold of 5.0. We reported discrimination with the C-statistic and 95% confidence intervals from 1,000 bootstrap resamples. Additional model fit statistics are reported in the Supplement.

### Outcomes and Complications

As a secondary aim, we compared clinical outcomes across three ventilation groups: no ventilation (0 days), brief ventilation (1 day), and prolonged ventilation (≥2 days). We kept the 1-day group separate rather than collapsing it into either adjacent category because a single ventilator day most often represents brief peri-procedural or precautionary intubation, whereas 2 or more days reflects sustained ventilatory support, and combining them would blur two clinically distinct courses. We defined in-hospital mortality by discharge disposition, ICU admission as total ICU days greater than 0, and prolonged ICU stay as 3 days or greater. We reported hospital and ICU length of stay as median with interquartile range. Using NTDB event codes, we assessed deep vein thrombosis, pulmonary embolism, pneumonia, ventilator-associated pneumonia, acute respiratory distress syndrome, sepsis, surgical site infection, and unplanned intubation. We compared binary outcomes with chi-squared tests and continuous outcomes with Kruskal-Wallis tests. We also calculated SMDs for the no-ventilation and prolonged-ventilation groups. Complications were compared on a cohort of the dataset (2019-2022) due to limitations in reporting and documentation in earlier years. Specifically, the NTDB introduced its structured hospital-events file, which records complications with standardized event codes, beginning with the 2019 data year; the 2017 and 2018 files captured complications under a different and noncomparable structure, so including them would have introduced coding heterogeneity.

We conducted eight prespecified sensitivity analyses yielding 14 subgroup models to assess robustness across fracture-level subgroups, developmental age groups, time periods, stricter ventilation definitions, complete-case analyses, missingness-indicator models, and PALS-based hypotension thresholds. We calculated E-values for clinically significant variables. Temperature had the highest missingness of any vital sign at 9.7%, and because hypothermia was itself a strong predictor we treated unrecorded temperature as nonhypothermic under our missing-equals-normal convention; the missingness-indicator and complete-case sensitivity analyses confirmed that this did not materially change the findings.

## Results

### Study Population and Mechanical Ventilation Prevalence

**Figure 1.**
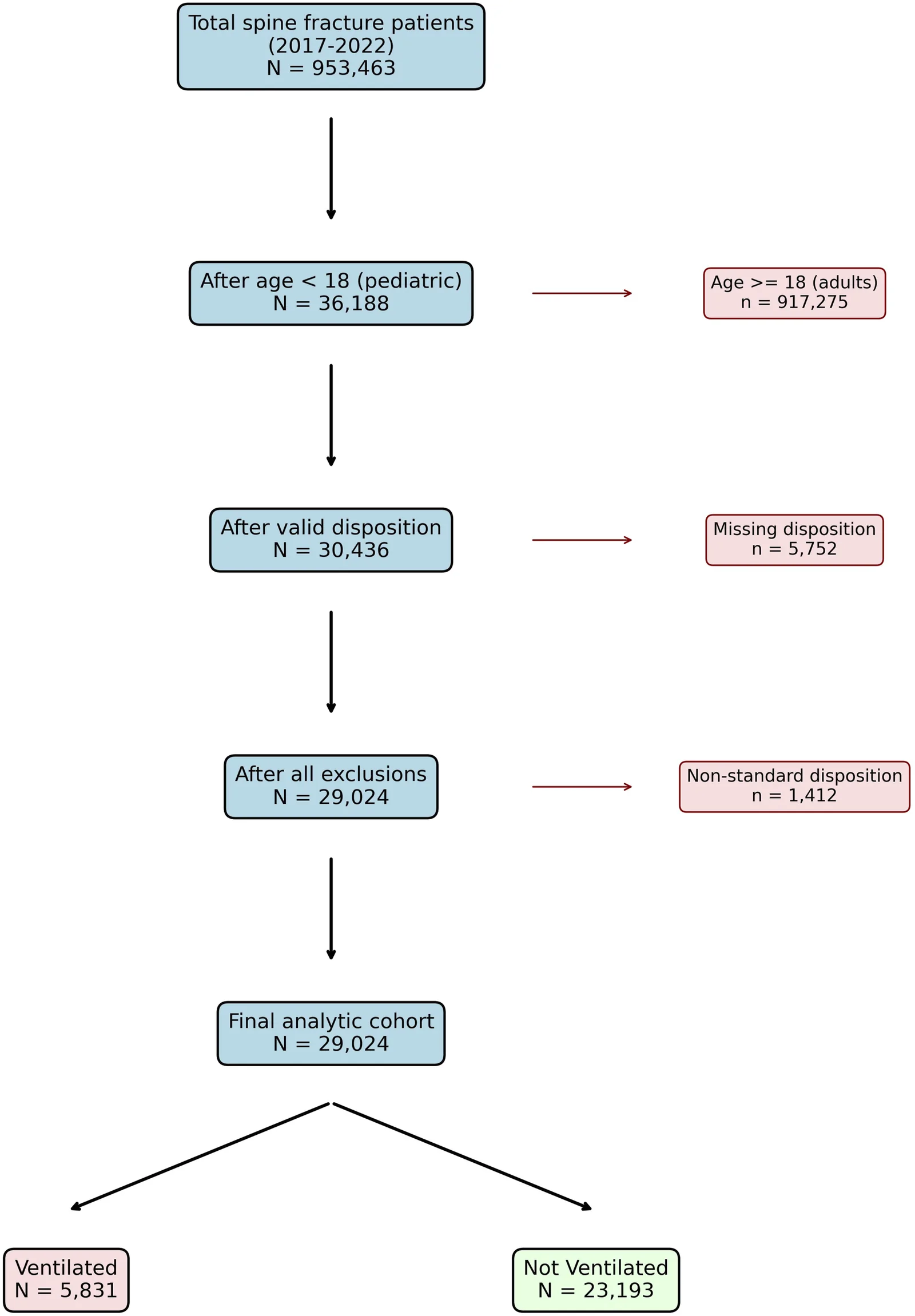
STROBE flow diagram. Of 953,463 spine fracture patients in the NTDB (2017-2022), 917,275 adults were excluded. After further exclusions for missing or non-standard disposition, 29,024 pediatric patients comprised the final cohort; 5,831 (20.1%) received mechanical ventilation.

We identified 953,463 patients with spine fractures in the NTDB (2017-2022). After excluding 917,275 adults (age ≥18 years), 36,188 pediatric patients remained. Exclusion of patients with missing discharge disposition and non-standard destinations yielded a final cohort of 29,024 patients (Figure 1). Of these, 5,831 (20.1%) received mechanical ventilation during the index hospitalization. Adolescents (13-17 years) comprised 74.4% of the cohort. Males accounted for 60.4% of patients and were overrepresented among ventilated patients (65.9% vs 59.0%). Motor vehicle collisions were the predominant mechanism (72.8%), and cervical fractures were present in 52.1% of patients (Table 1).

**Table 1.** Baseline Characteristics of Ventilated and Non-ventilated Pediatric Spine Fracture Patients

Variable	Overall	Ventilated	Not ventilated	SMD
	N (%)	N (%)	N (%)	
**Total**	29,024	5,831	23,193	​
Pediatric Age				
Infant (0-1 yr)	280 (1.0)	100 (1.7)	180 (0.8)	0.085
Toddler (2-5 yr)	1,789 (6.2)	543 (9.3)	1,246 (5.4)	0.151
School-age (6-12 yr) (reference)	5,370 (18.5)	1,011 (17.3)	4,359 (18.8)	-0.038
Adolescent (13-17 yr)	21,585 (74.4)	4,177 (71.6)	17,408 (75.1)	-0.077
Sex				
Female Sex	11,500 (39.6)	1,989 (34.1)	9,511 (41.0)	-0.143
Race/Ethnicity				
Hispanic Ethnicity	4,839 (16.7)	1,089 (18.7)	3,750 (16.2)	0.066
Black Race	4,752 (16.4)	1,262 (21.6)	3,490 (15.0)	0.171
Other Race/Ethnicity	1,869 (6.4)	424 (7.3)	1,445 (6.2)	0.042
ED Vitals				
Shock Index ≥ 0.9	9,489 (32.7)	3,094 (53.1)	6,395 (27.6)	0.538
SBP < 90 mmHg	1,253 (4.3)	881 (15.1)	372 (1.6)	0.503
RR < 12/min	486 (1.7)	289 (5.0)	197 (0.8)	0.246
RR > 20/min	10,763 (37.1)	2,599 (44.6)	8,164 (35.2)	0.192
SpO2 ≤ 92%	1,413 (4.9)	819 (14.0)	594 (2.6)	0.426
GCS 9-12	887 (3.1)	540 (9.3)	347 (1.5)	0.349
GCS ≤ 8	3,663 (12.6)	3,501 (60.0)	162 (0.7)	1.689
Hypothermic (<36 C)	1,590 (5.5)	1,033 (17.7)	557 (2.4)	0.526
Hyperthermic (>38 C)	466 (1.6)	163 (2.8)	303 (1.3)	0.105
Body Composition				
BMI < 18.5 (Underweight)	4,626 (15.9)	1,055 (18.1)	3,571 (15.4)	0.072
BMI Missing	5,385 (18.6)	944 (16.2)	4,441 (19.1)	-0.078
Mechanism				
MVC	21,130 (72.8)	4,446 (76.2)	16,684 (71.9)	0.099
Other Mechanism	2,395 (8.3)	857 (14.7)	1,538 (6.6)	0.264
Trauma Type				
Penetrating Trauma	1,442 (5.0)	622 (10.7)	820 (3.5)	0.280
Transport				
Private Vehicle	1,940 (6.7)	53 (0.9)	1,887 (8.1)	-0.353
Air Transport	6,727 (23.2)	2,500 (42.9)	4,227 (18.2)	0.555
Other Transport	147 (0.5)	57 (1.0)	90 (0.4)	0.072
Insurance				
Medicaid	10,590 (36.5)	2,384 (40.9)	8,206 (35.4)	0.113
Uninsured (Self-Pay)	1,659 (5.7)	349 (6.0)	1,310 (5.6)	0.014
Charity Care	42 (0.1)	6 (0.1)	36 (0.2)	-0.015
Substance Use				
Alcohol Positive	1,230 (4.2)	342 (5.9)	888 (3.8)	0.095
Toxicology Positive	3,760 (13.0)	1,077 (18.5)	2,683 (11.6)	0.194
Transfer Status				
Interfacility Transfer	11,664 (40.2)	1,948 (33.4)	9,716 (41.9)	-0.176
Fracture Level				
Thoracic Fracture	13,917 (47.9)	2,761 (47.4)	11,156 (48.1)	-0.015
Lumbar Fracture	13,282 (45.8)	2,290 (39.3)	10,992 (47.4)	-0.164
Protective Devices				
Child Restraint	660 (2.3)	217 (3.7)	443 (1.9)	0.110
Seatbelt	7,217 (24.9)	1,115 (19.1)	6,102 (26.3)	-0.172
Helmet	2,685 (9.3)	249 (4.3)	2,436 (10.5)	-0.240

*Note.* SMD = standardized mean difference; ED = emergency department; yr = years; SBP = systolic blood pressure; mmHg = millimeters of mercury; RR = respiratory rate; SpO_2_ = peripheral oxygen saturation; GCS = Glasgow Coma Scale; °C = degrees Celsius; BMI = body mass index; MVC = motor vehicle collision. Values are reported as N (%) unless otherwise indicated. An absolute SMD >0.10 was considered suggestive of meaningful imbalance between ventilated and non-ventilated patients. Reference categories were school-age children aged 6 to 12 years, male sex, White non-Hispanic race/ethnicity, normal vital signs, BMI 18.5 to 24.9, falls, blunt trauma, ground ambulance transport, commercial/private insurance, negative alcohol and toxicology screens, direct admission, cervical fracture, and absence of documented protective device use.

### Predictors of Mechanical Ventilation

**Figure 2.**
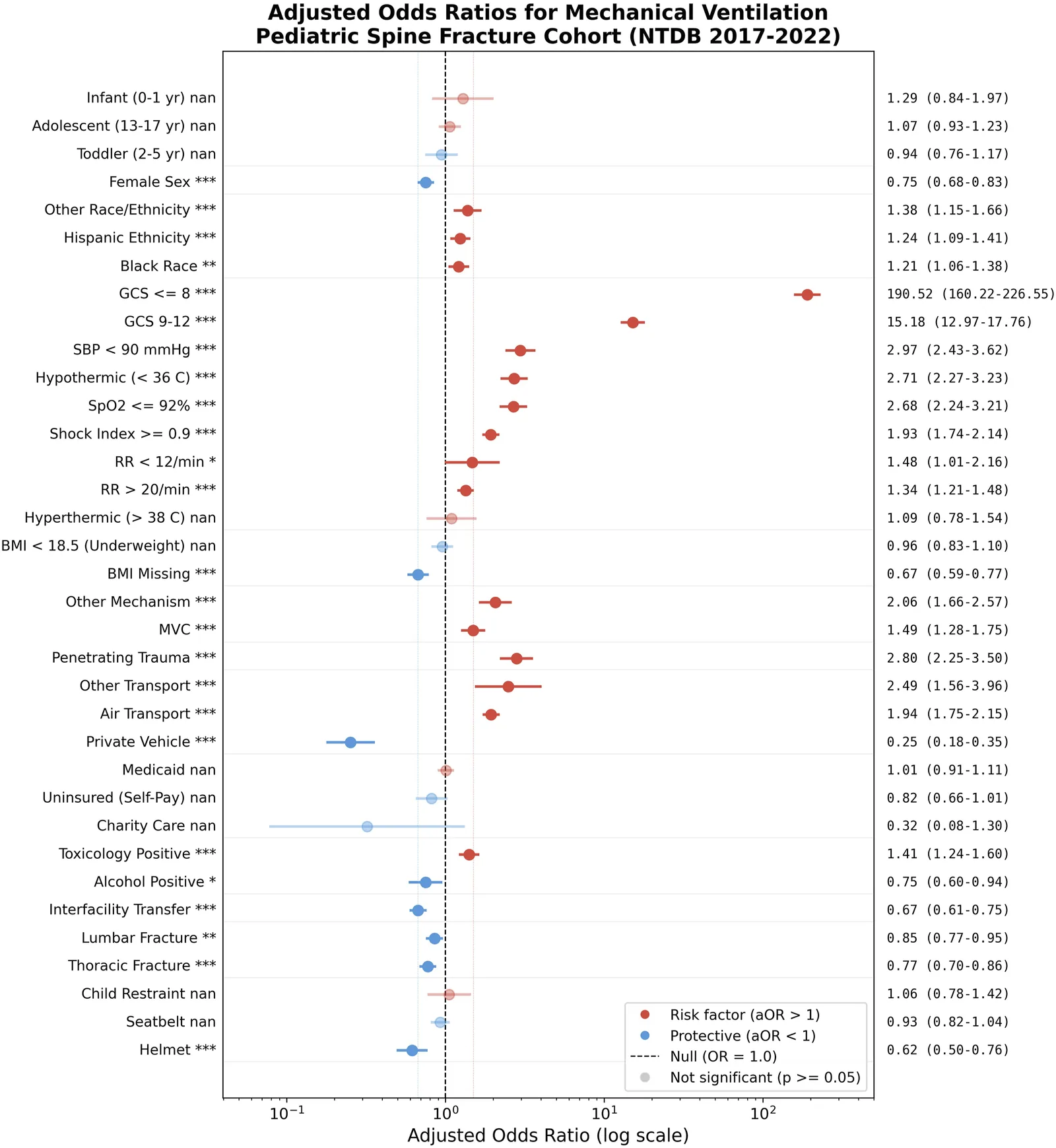
Forest plot of adjusted odds ratios from the primary multivariable logistic regression model, grouped by clinical domain. Red markers indicate risk factors (aOR > 1.0); blue markers indicate protective factors. Horizontal bars represent 95% confidence intervals. Logarithmic scale.

The primary multivariable model included 35 predictors and identified 18 variables meeting prespecified clinical significance thresholds (Table 2). Neurologic status was the dominant predictor domain. Compared with patients with normal mental status, those with Glasgow Coma Scale (GCS) ≤ 8 had markedly higher odds of ventilation (aOR 190.52; 95% CI 160.22-226.55), and GCS 9-12 also conferred substantially elevated odds (aOR 15.18; 95% CI 12.97-17.76). Among ventilated patients, 60.0% had GCS ≤ 8 compared with 0.7% of non-ventilated patients (SMD 1.689). Figure 2 visually displays the adjusted regression estimates of each predictor variable to visualize comparison of effect size, direction, and confidence intervals across covariates.

**Table 2.** Multivariable Logistic Regression: ED-Available Variables Associated With Mechanical Ventilation in Pediatric Spine Fracture Patients

Variable	aOR	95% CI	P value	Clinical significance
Pediatric Age				
Infant (0-1 yr)	1.29	0.84-1.97	0.247	​
Toddler (2-5 yr)	0.94	0.76-1.17	0.604	​
Adolescent (13-17 yr)	1.07	0.93-1.23	0.374	​
Sex				
Female Sex	0.75	0.68-0.83	<0.001***	​
Race/Ethnicity				
Hispanic Ethnicity	1.24	1.09-1.41	<0.001***	​
Black Race	1.21	1.06-1.38	0.004**	​
Other Race/Ethnicity	1.38	1.15-1.66	<0.001***	​
ED Vitals				
Shock Index ≥ 0.9	1.93	1.74-2.14	<0.001***	Risk
SBP < 90 mmHg	2.97	2.43-3.62	<0.001***	Risk
RR < 12/min	1.48	1.01-2.16	0.045*	​
RR > 20/min	1.34	1.21-1.48	<0.001***	​
SpO2 ≤ 92%	2.68	2.24-3.21	<0.001***	Risk
GCS 9-12	15.18	12.97-17.76	<0.001***	Risk
GCS ≤ 8	190.52	160.22-226.55	<0.001***	Risk
Hypothermic (<36 C)	2.71	2.27-3.23	<0.001***	Risk
Hyperthermic (>38 C)	1.09	0.78-1.54	0.613	​
Body Composition				
BMI < 18.5 (Underweight)	0.96	0.83-1.10	0.534	​
BMI Missing	0.67	0.59-0.77	<0.001***	​
Mechanism				
MVC	1.49	1.28-1.75	<0.001***	​
Other Mechanism	2.06	1.66-2.57	<0.001***	Risk
Trauma Type				
Penetrating Trauma	2.80	2.25-3.50	<0.001***	Risk
Transport				
Private Vehicle	0.25	0.18-0.35	<0.001***	Protective
Air Transport	1.94	1.75-2.15	<0.001***	Risk
Other Transport	2.49	1.56-3.96	<0.001***	Risk
Insurance				
Medicaid	1.01	0.91-1.11	0.902	​
Uninsured (Self-Pay)	0.82	0.66-1.01	0.065	​
Charity Care	0.32	0.08-1.30	0.112	Protective
Substance Use				
Alcohol Positive	0.75	0.60-0.94	0.013*	​
Toxicology Positive	1.41	1.24-1.60	<0.001***	​
Transfer Status				
Interfacility Transfer	0.67	0.61-0.75	<0.001***	​
Fracture Level				
Thoracic Fracture	0.77	0.70-0.86	<0.001***	​
Lumbar Fracture	0.85	0.77-0.95	0.003**	​
Protective Devices				
Child Restraint	1.06	0.78-1.42	0.718	​
Seatbelt	0.93	0.82-1.04	0.207	​
Helmet	0.62	0.50-0.76	<0.001***	Protective

*Note.* aOR = adjusted odds ratio; CI = confidence interval; ED = emergency department; yr = years; SBP = systolic blood pressure; mmHg = millimeters of mercury; RR = respiratory rate; SpO_2_ = peripheral oxygen saturation; GCS = Glasgow Coma Scale; °C = degrees Celsius; BMI = body mass index; MVC = motor vehicle collision; N = number of patients. Model C-statistic was 0.928 (95% CI, 0.924-0.932), and McFadden pseudo-R^2^ was 0.541. The analytic cohort included 29,024 patients, of whom 5,831 (20.1%) received mechanical ventilation. Clinical significance was defined as aOR >1.50 for risk factors and aOR <0.67 for protective factors. Reference categories were school-age children aged 6 to 12 years, male sex, White non-Hispanic race/ethnicity, normal vital signs, BMI 18.5 to 24.9, falls, blunt trauma, ground ambulance transport, commercial/private insurance, negative alcohol and toxicology screens, direct admission, cervical fracture, and absence of documented protective device use. *P < .05; **P < .01; ***P < .001.

Hemodynamic instability markers were independently associated with ventilation: hypotension (aOR 2.97; 95% CI 2.43-3.62), hypothermia (aOR 2.71; 95% CI 2.27-3.23), and shock index ≥ 0.9 (aOR 1.93; 95% CI 1.74-2.14). Respiratory compromise was also predictive, including hypoxemia with SpO_2_ ≤ 92% (aOR 2.68; 95% CI 2.24-3.21), bradypnea with RR < 12 (aOR 1.48; 95% CI 1.01-2.16), and tachypnea with RR > 20 (aOR 1.34; 95% CI 1.21-1.48). Among injury characteristics, penetrating trauma (aOR 2.80; 95% CI 2.25-3.50) was a significant risk factor, while thoracic (aOR 0.77; 95% CI 0.70-0.86) and lumbar (aOR 0.85; 95% CI 0.77-0.95) fractures were protective relative to cervical fractures. Air transport was associated with increased odds (aOR 1.94; 95% CI 1.75-2.15), whereas private vehicle arrival (aOR 0.25; 95% CI 0.18-0.35), helmet use (aOR 0.62; 95% CI 0.50-0.76), interfacility transfer (aOR 0.67), and female sex (aOR 0.75) were protective.

### Model Performance and Robustness

**Figure 3.**
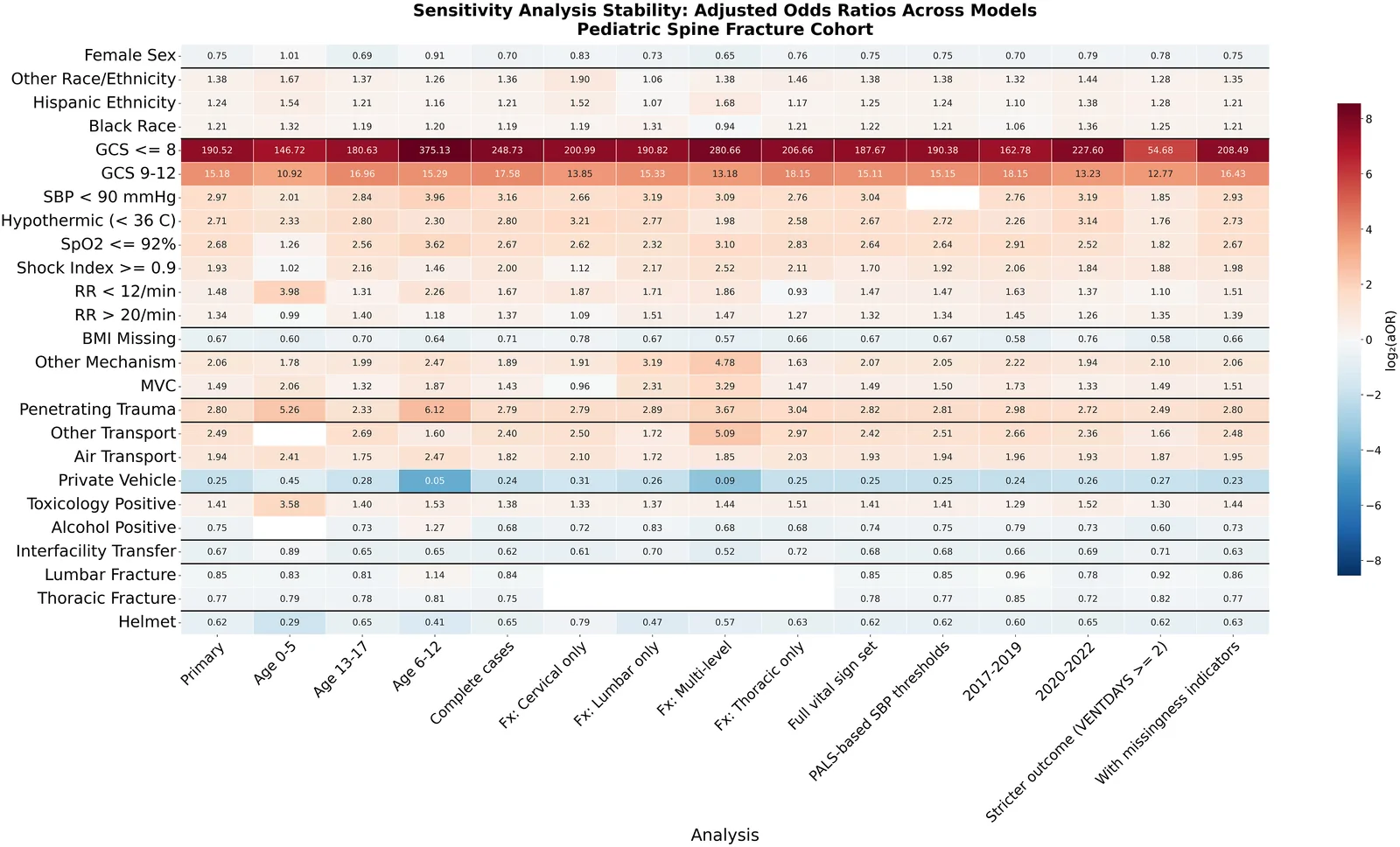
Sensitivity analysis heatmap showing adjusted odds ratios across the primary model and 13 sensitivity analyses. Color intensity reflects log₂-transformed aOR on a diverging scale: red = risk, blue = protection, white = null. Variables limited to those significant (p < 0.05) in the primary model

The model showed strong discrimination (C-statistic 0.928; 95% CI 0.924-0.932; McFadden pseudo-R^2^ = 0.541) with no concerning multicollinearity (all VIFs < 2.1; maximum 2.06 for other mechanism; Supplemental Table S1). Across 14 subgroup and sensitivity analyses, the model demonstrated consistently excellent discrimination (C-statistics, 0.916-0.940). GCS ≤ 8 remained the strongest independent predictor of mechanical ventilation across all analytic variations, including fracture-level, developmental age, temporal, alternative-definition, missing-data, and PALS-based hypotension analyses (aOR range, 54.68-375.13; Table 3). Figure 3 visually summarizes the stability of adjusted associations across the primary and sensitivity models for all predictors that were significant in the primary model. E-values supported robustness to unmeasured confounding (GCS ≤ 8 E-value 380.54, lower bound 319.95; Supplemental Table S2). Most ED variables had < 5% missingness; temperature had the highest at 9.7% (Supplemental Table S3). Results were consistent in complete-case and missingness-indicator analyses.

**Table 3.** Sensitivity Analyses Summary

Analysis	N	Events	C-statistic	OR GCS ≤ 8	OR SI ≥ 0.9	OR hypoxic	OR infant	OR air transport	OR thoracic Fx
Full vital sign set	29,024	5,831	0.928	187.67	1.70	2.64	1.26	1.93	0.78
Fracture: Cervical only	5,108	1,393	0.933	200.99	1.12	2.62	1.25	2.10	​
Fracture: Thoracic only	10,634	2,148	0.940	206.66	2.11	2.83	1.80	2.03	​
Fracture: Lumbar only	9,999	1,677	0.916	190.82	2.17	2.32	0.73	1.72	​
Fracture: Multi-level	3,283	613	0.928	280.66	2.52	3.10	0.11	1.85	​
Period: 2017-2019	13,336	2,716	0.931	162.78	2.06	2.91	0.99	1.96	0.85
Period: 2020-2022	15,688	3,115	0.926	227.60	1.84	2.52	1.66	1.93	0.72
Stricter outcome (total ventilation days ≥ 2)	29,024	5,081	0.918	54.68	1.88	1.82	1.18	1.87	0.82
Age group: Age 0-5	2,069	643	0.931	146.72	1.02	1.26	​	2.41	0.79
Age group: Age 6-12	5,370	1,011	0.940	375.13	1.46	3.62	​	2.47	0.81
Age group: Age 13-17	21,585	4,177	0.926	180.63	2.16	2.56	​	1.75	0.78
Complete cases	24,565	4,348	0.930	248.73	2.00	2.67	1.26	1.82	0.75
With missingness indicators	29,024	5,831	0.932	208.49	1.98	2.67	1.19	1.95	0.77
PALS-based SBP thresholds	29,024	5,831	0.928	190.38	1.92	2.64	1.40	1.94	0.77

*Note.* N = number of patients; OR = odds ratio; GCS = Glasgow Coma Scale; SI = shock index; hypoxic = SpO_2_ ≤92%; SpO_2_ = peripheral oxygen saturation; Fx = fracture; PALS = Pediatric Advanced Life Support; SBP = systolic blood pressure. The primary model had a C-statistic of 0.928 in 29,024 patients, with 5,831 ventilation events, and an OR of 190.52 for GCS ≤8. All sensitivity analyses used full multivariable logistic regression with the same analytic approach as the primary model. Fracture-level subgroup analyses excluded fracture-level predictors, and age-group subgroup analyses excluded age-group predictors. The PALS-based analysis replaced the universal SBP <90 mmHg threshold with age-specific hypotension thresholds. Blank cells indicate predictors that were not included, not estimable, or not applicable within that subgroup model.

### Clinical Burden of Mechanical Ventilation

Among 29,024 patients, 23,193 (79.9%) had no ventilation, 750 (2.6%) had 1 ventilator day, and 5,081 (17.5%) had ≥ 2 ventilator days (Table 4). In-hospital mortality differed substantially across groups (0.20%, 27.9%, and 15.7%, respectively; p<0.001). The higher mortality in the 1-day group than in the group ventilated for 2 or more days most likely reflects a survivorship pattern rather than any protective effect of longer ventilation, because the 1-day group is enriched for children who were intubated but died within the first day, before ventilation could be continued. ICU admission rates increased as ventilation usage increased (no ventilation: 31.3%, 1 ventilator day: 90.1%, and ≥ 2 ventilator days: 99.0%), as did prolonged ICU stays of ≥ 3 days (17.2%, 39.3%, and 90.2% respectively). Median hospital length of stay was 3, 5, and 15 days, respectively.

**Table 4.** Outcomes and Complications by Ventilation Group (NTDB 2017-2022)

Outcome	No ventilation (n = 23,193)	1 vent day (n = 750)	≥ 2 vent days (n = 5,081)	P value	SMD (0 vs ≥ 2)
Outcomes					
In-hospital mortality	46 (0.20%)	209 (27.9%)	798 (15.7%)	<0.001	0.597
ICU admission	7,259 (31.3%)	676 (90.1%)	5,030 (99.0%)	<0.001	2.023
Prolonged ICU (≥3 days)	3,989 (17.2%)	295 (39.3%)	4,583 (90.2%)	<0.001	2.149
ICU days, median [IQR]	0 [0-2]	2 [1-4]	7 [4-15]	<0.001	1.094
Hospital LOS, median [IQR]	3 [2-6]	5 [2-10]	15 [8-26]	<0.001	1.018
Complications					
Deep vein thrombosis	32 (0.20%)	2 (0.40%)	126 (3.6%)	<0.001	0.25
Pulmonary embolism	11 (0.07%)	1 (0.20%)	35 (1.0%)	<0.001	0.128
DVT or PE	42 (0.26%)	3 (0.60%)	154 (4.4%)	<0.001	0.278
Ventilator-associated pneumonia	0 (0.00%)	4 (0.80%)	193 (5.5%)	<0.001	0.321
ARDS	0 (0.00%)	1 (0.20%)	81 (2.3%)	<0.001	0.217
Sepsis	16 (0.10%)	4 (0.80%)	175 (5.0%)	<0.001	0.318
Wound infection/SSI	32 (0.20%)	3 (0.60%)	168 (4.8%)	<0.001	0.294

*Note.* Values are reported as N (%) for binary outcomes and median [IQR] for continuous outcomes. NTDB = National Trauma Data Bank; n = number of patients; ICU = intensive care unit; LOS = length of stay; IQR = interquartile range; SMD = standardized mean difference; DVT = deep vein thrombosis; PE = pulmonary embolism; VAP = ventilator-associated pneumonia; ARDS = acute respiratory distress syndrome; SSI = surgical site infection; PUF = Participant Use File. P values were calculated using chi-square tests for binary outcomes and Kruskal-Wallis tests for continuous outcomes. SMD compares patients with no ventilation versus patients with ≥2 ventilator days. Complication data were available only from 2019 to 2022; therefore, complication rows reflect the 2019-2022 subset. Outcome rows, including mortality, ICU admission, ICU days, and hospital LOS, reflect the full 2017-2022 cohort. “DVT or PE” reports the number of unique patients with either event and is therefore not the arithmetic sum of the separate DVT and PE rows.

In the complications subset (2019-2022; n=20,133), prolonged ventilation was associated with marked increases in all measured complications: sepsis (no ventilation: 0.1% vs 1 ventilator day: 0.8% vs ≥ 2 ventilator days: 5.0%), DVT or PE (0.3%, 0.6%, and 4.4% respectively), ventilator-associated pneumonia (0.0%, 0.8%, and 5.5% respectively), and ARDS (0.0%, 0.2%, and 2.3% respectively; all p<0.001).

## Discussion

In this national cohort of 29,024 pediatric patients with spine fractures, one in five required mechanical ventilation. Several emergency department variables independently identified children at increased risk, with severe neurologic impairment emerging as the dominant predictor. Patients presenting with GCS scores of 8 or below had 190-fold higher odds of mechanical ventilation than those with normal mental status. Elevated shock index, hypotension, hypothermia, hypoxemia, abnormal respiratory rates, cervical fracture location, and penetrating trauma also independently predicted ventilation. Prolonged mechanical ventilation was associated with substantial morbidity and mortality, including a 15.7% mortality rate and higher rates of sepsis, venous thromboembolism, and ventilator-associated pneumonia.

The magnitude of the association between depressed mental status and mechanical ventilation exceeds that reported in many adult trauma studies and likely reflects the particular vulnerability of children with spine trauma.^15,16,18^ GCS of 8 or below remains the accepted threshold for severe traumatic brain injury and loss of airway protective reflexes in both adult and pediatric practice.^18,19^ In our cohort, 60% of ventilated patients had GCS ≤ 8 at presentation, suggesting that associated traumatic brain injury drives many early ventilation decisions in pediatric spine trauma. This interpretation is consistent with pediatric head injury literature linking lower GCS to higher risks of clinically important traumatic brain injury, neurosurgical intervention, and mortality.^20-22^ More broadly, these findings extend prior pediatric spine trauma literature, which has identified younger age, upper cervical injury, and spinal cord injury as risk factors for poor outcomes, but has not specifically examined mechanical ventilation using emergency department variables available at presentation.^1,4,5^ Adult spinal cord injury studies similarly show that neurologic level and completeness of injury predict respiratory failure, although direct extrapolation to children is limited by differences in spinal anatomy and physiology.^2,3,15,16,23^

The other major predictors were also biologically plausible. High cervical spinal cord injury can impair phrenic nerve function at C3-C5, while lower cervical and upper thoracic injuries weaken intercostal muscle function and reduce effective ventilation.^3,15,16^ Hypotension may reflect hemorrhagic shock, neurogenic shock, or both, all of which can prompt intubation to facilitate resuscitation and reduce secondary brain injury from hypoperfusion.^18,24^ Hypothermia likely marks severe physiologic stress and contributes to the trauma lethal triad.^25,26^ Shock index has emerged as a useful pediatric trauma triage tool for predicting transfusion, intensive care admission, and mortality,^27-29^ and in our cohort shock index ≥ 0.9 remained independently associated with mechanical ventilation even after adjustment for hypotension and other hemodynamic variables.^30^ This suggests that it may capture physiologic instability and poor tissue perfusion before overt hypotension develops.^27,28,31^

The multivariate model performed well, with excellent discrimination (C-statistic 0.928), and remained stable across 14 subgroup sensitivity analyses spanning fracture-levels, developmental age, time periods, and missing data strategies. GCS ≤ 8 remained the strongest predictor in every subgroup, with adjusted odds ratios ranging from 54.68 to 375.13. E-value analysis further strengthened confidence in this result, as an unmeasured confounder would need an odds ratio greater than 380 with both GCS ≤ 8 and mechanical ventilation to fully explain the finding. Although age-stratified analyses suggested developmental variation, with the highest adjusted odds ratio for GCS ≤ 8 seen in school-age children, these subgroup differences should be interpreted cautiously.^6,7,13^

These findings have practical implications for spine and trauma surgeons. At presentation, readily available factors such as GCS, shock index, and cervical fracture location can help to identify children at high risk for ventilation, facilitating early airway planning, aggressive resuscitation, and transfer to centers with pediatric neurosurgical and critical care resources. The results may also inform trauma activation, since current American College of Surgeons pediatric activation criteria have limited sensitivity for severe injury, and incorporation of shock index and mental status may improve triage.^32^ In addition, these findings may help guide early discussions with families regarding likely ventilator needs and expected hospital courses. The high complication burden associated with prolonged ventilation also reinforces the importance of lung-protective ventilation and early mobilization when feasible.^10,12,33^

Helmet use was the only potentially modifiable risk factor identified. Its association with a 38% lower risk of mechanical ventilation is consistent with evidence that helmets reduce traumatic brain injury and cervical spine injury severity in motorcycle, bicycle, and recreational vehicle crashes.^34-38^ Although causation cannot be inferred, this finding supports continued helmet legislation and injury prevention efforts, areas in which spine surgeons and trauma teams can play an important advocacy role.^4^

Hispanic ethnicity, Black race, and other race or ethnicity were each independently associated with higher odds of mechanical ventilation (adjusted odds ratios 1.24, 1.21, and 1.38, respectively). These associations should be interpreted with caution. They may reflect differential care, unmeasured differences in injury severity or physiologic presentation not captured by the available emergency department covariates, or the recognized limitations of race and ethnicity coding in the National Trauma Data Bank. Persistent racial, ethnic, and socioeconomic disparities in trauma outcomes have been documented across the continuum of care.^39^

This study has several strengths. To our knowledge, it is the largest analysis of mechanical ventilation predictors in pediatric spine fractures. The large national sample yielded a strong events-per-variable ratio, reducing risk of overfitting, and the focus on emergency department variables makes the model clinically useful at triage. The use of familiar thresholds, including GCS ≤ 8 and shock index ≥ 0.9, further supports bedside applicability.

This study also has important limitations. The NTDB does not record prehospital intubation status, so the outcome includes children intubated before emergency department arrival, and some predictors may therefore partly reflect prehospital decision-making rather than in-hospital deterioration. Because a low Glasgow Coma Scale score and hypoxemia may partly capture the pre-intubation state of children who arrived already ventilated, the associations between these predictors and the ventilation outcome are partly circular and most likely overstate their true independent effect, so readers should regard these particular estimates as upper bounds. Residual confounding remains possible in any retrospective registry analysis, although the E-value findings suggest that the primary GCS result is robust. The missing-equals-normal approach for vital signs may have introduced misclassification, though alternative missing data analyses were consistent. Spinal cord injury status, likely one of the most clinically relevant predictors of ventilation, was not reliably coded and therefore could not be included.^15,16^ This is an important consideration because spinal cord injury directly affects respiratory function and may mediate part of the association between cervical fracture location and ventilation. Cervical fracture location may therefore act partly as a proxy for undetected cord injury, so its independent anatomical contribution to ventilation risk may be smaller than our estimates suggest. Finally, NTDB ICD-10 coding captures the level of vertebral fracture but not its morphologic subtype, so we could not assign an AO Spine classification, which requires imaging and operative detail the registry does not record; we therefore analyzed fracture level without fracture morphology. Prospective multicenter validation with prehospital data, spinal cord injury classification, and longitudinal outcomes is needed.

## Conclusion

In this national cohort of pediatric spine fracture patients, GCS, hemodynamic instability, respiratory compromise, and cervical fracture location were the strongest emergency department predictors of mechanical ventilation. Prolonged ventilation was associated with 15.7% mortality and substantially higher complication rates, highlighting the value of early risk identification. These findings may support early triage, airway planning, and resource allocation in pediatric spine trauma. Prospective validation studies that incorporate prehospital data and spinal cord injury classification are needed to refine these estimates and determine their clinical impact.

## Supplemental Material

Supplemental material - Emergency Department Predictors of Mechanical Ventilation in Pediatric Spine Fracture Patients in the US Supplemental material for Emergency Department Predictors of Mechanical Ventilation in Pediatric Spine Fracture Patients in the US by Samer G. Salman, Rohan A. Phadke, Margaret A. Baldwin in Global Spine Journal

## Data Availability

Upon request.*

## References

[1] Shin JI Lee NJ Cho SK . Pediatric cervical spine and spinal cord injury: a national database study. Spine (Phila Pa 1976). 2016;41(4):283-292. doi:10.1097/BRS.0000000000001176. 26555836

[2] McAllister AS Nagaraj U Radhakrishnan R . Emergent imaging of pediatric cervical spine trauma. Radiographics. 2019;39(4):1126-1142. doi:10.1148/rg.2019180100. 31173542

[3] Klimo PJ Ware ML Gupta N Brockmeyer D . Cervical spine trauma in the pediatric patient. Neurosurg Clin N Am. 2007;18(4):599-620. doi:10.1016/j.nec.2007.09.004. 17991586

[4] García-Rudolph A Wright MA Rivas N Opisso E Vidal J . Epidemiology of pediatric spinal trauma with neurological deficits in Catalonia: a 36-year experience. Eur Spine J. 2024;33(12):4437-4448. doi:10.1007/s00586-024-08351-1. 38852115

[5] Falavigna A Righesso O Guarise da Silva P , et al. Epidemiology and management of spinal trauma in children and adolescents <18 years old. World Neurosurg. 2018;110:e479-e483. doi:10.1016/j.wneu.2017.11.021. 29146435

[6] Alas H Pierce KE Brown A , et al. Sports-related cervical spine fracture and spinal cord injury: a review of nationwide pediatric trends. Spine (Phila Pa 1976). 2021;46(1):22-28. doi:10.1097/BRS.0000000000003718. 32991512

[7] Compagnon R Ferrero E Leroux J , et al. Epidemiology of spinal fractures in children: cross-sectional study. Orthop Traumatol Surg Res. 2020;106(7):1245-1249. doi:10.1016/j.otsr.2020.06.015. 33060015

[8] Correia MC Gonçalves Santos D Relvas SM , et al. Paediatric cervical spine injuries: a descriptive analysis of thirty-two years of experience at a trauma centre. Int Orthop. 2025;50:213-218. doi:10.1007/s00264-025-06727-6. Published online. 41402532

[9] Reitano E Gavelli F Iannantuoni G , et al. In-hospital predictors of need for ventilatory support and mortality in chest trauma: a multicenter retrospective study. J Clin Med. 2023;12(2):714. doi:10.3390/jcm12020714. 36675639 PMC9863024

[10] Sood S Ganatra HA Perez Marques F Langner TR . Complications during mechanical ventilation: a pediatric intensive care perspective. Front Med (Lausanne). 2023;10:1016316. doi:10.3389/fmed.2023.1016316. 36817772 PMC9928727

[11] Principi T Fraser DD Morrison GC , et al. Complications of mechanical ventilation in the pediatric population. Pediatr Pulmonol. 2011;46(5):452-457. doi:10.1002/ppul.21389. 21194139

[12] Egbuta C Easley RB . Update on ventilation management in the pediatric intensive care unit. Paediatr Anaesth. 2022;32(2):354-362. doi:10.1111/pan.14374. 34882910

[13] Ruiz C Aquevedo A Marambio-Coloma C Arqueros L Bugedo G Damiani LF . Mechanical ventilation in trauma-induced respiratory failure: a clinical review. Respir Care. 2026;71:405-416. doi:10.1177/19433654261428443. Published online. 41823213

[14] Becker ER Wetmore GC Goodman MD Rodriquez D Branson RD . Review of ventilation in traumatic brain injury. Respir Care. 2025;70(4):450-457. doi:10.1089/respcare.12796. 40028858 PMC13178954

[15] Killien EY Mills B Watson RS Vavilala MS Rivara FP . Risk factors on hospital arrival for acute respiratory distress syndrome following pediatric trauma. Crit Care Med. 2018;46(12):e1088-e1096. doi:10.1097/CCM.0000000000003379. 30119074 PMC6239886

[16] Moulton AW Schauer SG Borgman MA . Prolonged mechanical ventilation in pediatric trauma patients in a combat zone. Pediatr Crit Care Med. 2022;23(12):1009-1016. doi:10.1097/PCC.0000000000003050. 35997515

[17] Committee on Trauma, American College of Surgeons. TQP PUF 2017.1.0; 2018.1.0; 2019.1.0; 2020.1.0; 2021.1.0; 2022.1.0. Chicago, IL: American College of Surgeons; 2017-2022.

[18] American College of Surgeons . Best Practices in the Management of Traumatic Brain Injury. Chicago, IL, United States: American College of Surgeons; 2024

[19] Lulla A Lumba-Brown A Totten AM , et al. Prehospital guidelines for the management of traumatic brain injury: 3rd edition. Prehosp Emerg Care. 2023;27(5):507-538. doi:10.1080/10903127.2023.2187905. 37079803

[20] Kochar A Borland ML Phillips N , et al. Association of clinically important traumatic brain injury and Glasgow Coma Scale scores in children with head injury. Emerg Med J. 2020;37(3):127-134. doi:10.1136/emermed-2018-208154. 32051126

[21] Johnson MA Nishijima DK Kuppermann N . The association of Glasgow Coma Scale score with clinically important traumatic brain injuries in children. Pediatr Emerg Care. 2020;36(11):e610-e613. doi:10.1097/PEC.0000000000001701. 32484321

[22] Cicero MX Cross KP . Predictive value of initial Glasgow Coma Scale score in pediatric trauma patients. Pediatr Emerg Care. 2013;29(1):43-48. doi:10.1097/PEC.0b013e31827b52bf. 23283262

[23] Kumaresan S Yoganandan N Pintar FA Maiman DJ Kuppa S . Biomechanical study of pediatric human cervical spine: a finite element approach. J Biomech Eng. 2000;122(1):60-71. doi:10.1115/1.429628. 10790831

[24] Picetti E Catena F Abu-Zidan F , et al. Early management of isolated severe traumatic brain injury patients in a hospital without neurosurgical capabilities: a consensus and clinical recommendations of the World Society of Emergency Surgery (WSES). World J Emerg Surg. 2023;18(1):5. doi:10.1186/s13017-022-00468-2. 36624517 PMC9830860

[25] de Reuver S Costa L van Rheenen H , et al. Disc and vertebral body morphology from birth to adulthood. Spine (Phila Pa 1976). 2022;47(7):E312-E318. doi:10.1097/BRS.0000000000004278. 34798645

[26] Charles YP Lamas V Ilharreborde B , et al. Comparison of spinopelvic configuration and Roussouly alignment types between pediatric and adult populations. Spine (Phila Pa 1976). 2022;47(18):1303-1313. doi:10.1097/BRS.0000000000004411. 35797644

[27] Yoon SH Shin SJ Kim H Roh YH . Shock index and shock index, pediatric age-adjusted as predictors of mortality in pediatric patients with trauma: a systematic review and meta-analysis. PLoS One. 2024;19(7):e0307367. doi:10.1371/journal.pone.0307367. 39024206 PMC11257222

[28] Georgette N Keskey R Hampton D , et al. Derivation and validation of an improved pediatric shock index for predicting need for early intervention and outcomes in pediatric trauma. J Trauma Acute Care Surg. 2022;93(4):474-481. doi:10.1097/TA.0000000000003727. 35749746

[29] Barone-Camp A Myers EK Bensard DD Acker SN . Shock index, pediatric age adjusted: a scoping review of applications in pediatric trauma triage and beyond. J Trauma Acute Care Surg. 2025;99(1):143-148. doi:10.1097/TA.0000000000004601. 40128162

[30] Hurley SP Soper B Pierce AE Gaccione A Burjonrappa SC . Critical shock index, pediatric age-adjusted as a predictive statistic for ICU admission in pediatric trauma. J Pediatr Surg. 2025;61:162555. doi:10.1016/j.jpedsurg.2025.162555. Published online. 40812410

[31] Georgette N Keskey R Mbadiwe N Hampton D McQueen A Slidell MB . Alternative shock index cutoffs for pediatric patients outperform the shock index pediatric age-adjusted (SIPA) on strength of association with adverse outcomes in pediatric trauma patients. Surgery. 2022;172(1):343-348. doi:10.1016/j.surg.2022.01.028. 35210102

[32] McCormick T Haukoos J Hopkins E , et al. Adding age-adjusted shock index to the American College of Surgeons' trauma team activation criteria to predict severe injury in children. J Trauma Acute Care Surg. 2023;94(2):295-303. doi:10.1097/TA.0000000000003693. 36694336

[33] Klompas M Branson R Cawcutt K , et al. Strategies to prevent ventilator-associated pneumonia, ventilator-associated events, and nonventilator hospital-acquired pneumonia in acute-care hospitals: 2022 update. Infect Control Hosp Epidemiol. 2022;43(6):687-713. doi:10.1017/ice.2022.88. 35589091 PMC10903147

[34] Ratnasekera AM Seng SS Gardiner SK , et al. Systematic review and meta-analysis of efficacy of helmet use and helmet laws to reduce mortality and cervical spine injury in adult motorcycle riders: a practice management guideline from the Eastern Association for the Surgery of Trauma. J Trauma Acute Care Surg. 2025;99:650-663. doi:10.1097/TA.0000000000004607. Published online. 40462278

[35] Park GJ Shin J Kim SC , et al. Protective effect of helmet use on cervical injury in motorcycle crashes: a case-control study. Injury. 2019;50(3):657-662. doi:10.1016/j.injury.2019.01.030. 30765183

[36] Page PS Wei Z Brooks NP . Motorcycle helmets and cervical spine injuries: a 5-year experience at a level 1 trauma center. J Neurosurg Spine. 2018;28(6):607-611. doi:10.3171/2017.7.SPINE17540. 29506463

[37] Page PS Burkett DJ Brooks NP . Association of helmet use with traumatic brain and cervical spine injuries following bicycle crashes. Br J Neurosurg. 2020;34(3):276-279. doi:10.1080/02688697.2020.1731425. 32106719

[38] Hooten KG Murad GJ . Helmet use and cervical spine injury: a review of motorcycle, moped, and bicycle accidents at a level 1 trauma center. J Neurotrauma. 2014;31(15):1329-1333. doi:10.1089/neu.2013.3253. 24661125

[39] Haider AH Weygandt PL Bentley JM , et al. Disparities in trauma care and outcomes in the United States: a systematic review and meta-analysis. J Trauma Acute Care Surg. 2013;74(5):1195-1205. doi:10.1097/TA.0b013e31828c331d. 23609267 PMC3641534

