# Supplementary material for: Emergency Department Predictors of Mechanical Ventilation in Pediatric Spine Fracture Patients in the US
Source: Global Spine J. 2026 Aug 24:21925682261483531. Online ahead of print. doi: 10.1177/21925682261483531 (PMC13506988; doi:10.1177/21925682261483531)
Supplement: Supplementary file 1 [file sj-pdf-1-gsj-10.1177_21925682261483531.pdf]

**Supplemental Materials**

**Supplemental Table S1. Variance inflation factors (VIF) for all predictor variables in the primary multivariable model.**

| **Variable** | **VIF** |
|---|---|
| Other Mechanism | 2.06 |
| Penetrating Trauma | 1.81 |
| MVC | 1.69 |
| Adolescent (13-17 yr) | 1.65 |
| Toddler (2-5 yr) | 1.42 |
| GCS ≤ 8 | 1.31 |
| Shock Index ≥ 0.9 | 1.30 |
| Lumbar Fracture | 1.28 |
| BMI < 18.5 (Underweight) | 1.28 |
| Black Race | 1.26 |
| Seatbelt | 1.26 |
| Thoracic Fracture | 1.25 |
| Child Restraint | 1.18 |
| RR > 20/min | 1.17 |
| Medicaid | 1.16 |
| SBP < 90 mmHg | 1.15 |
| Helmet | 1.15 |
| Private Vehicle | 1.13 |
| Hispanic Ethnicity | 1.13 |
| Air Transport | 1.12 |
| Toxicology Positive | 1.11 |
| Hypothermic (< 36 C) | 1.11 |
| Interfacility Transfer | 1.11 |
| BMI Missing | 1.11 |
| Infant (0-1 yr) | 1.09 |
| Female Sex | 1.08 |
| SpO2 ≤ 92% | 1.08 |
| Uninsured (Self-Pay) | 1.05 |
| Alcohol Positive | 1.05 |
| Other Race/Ethnicity | 1.05 |
| RR < 12/min | 1.04 |
| GCS 9-12 | 1.03 |
| Hyperthermic (> 38 C) | 1.02 |
| Other Transport | 1.02 |
| Charity Care | 1.00 |

Note. VIF = variance inflation factor; MVC = motor vehicle collision; GCS = Glasgow Coma Scale; RR = respiratory rate; SBP = systolic blood pressure; BMI = body mass index; $SpO_2$ = peripheral oxygen saturation; yr = years; °C = degrees Celsius; mmHg = millimeters of mercury. A VIF >5 was considered suggestive of moderate multicollinearity, and a VIF >10 was considered suggestive of severe

multicollinearity. The maximum VIF was 2.06 for Other Mechanism, indicating no concerning multicollinearity among predictor variables in the primary multivariable model.

**Supplemental Table S2. E-Value Sensitivity Analysis for Unmeasured Confounding for all clinically significant variables (aOR > 1.5 or < 0.67 with $p < 0.05$).**

| Variable | aOR | 95% CI | P Value | E-value (Point) | E-value (CI Limit) | Direction |
|---|---|---|---|---|---|---|
| Shock Index ≥ 0.9 | 1.93 | (1.74-2.14) | <0.001 | 3.27 | 2.87 | Risk |
| SBP < 90 mmHg | 2.97 | (2.43-3.62) | <0.001 | 5.38 | 4.29 | Risk |
| SpO2 ≤ 92% | 2.68 | (2.24-3.21) | <0.001 | 4.80 | 3.90 | Risk |
| GCS 9-12 | 15.18 | (12.97-17.76) | <0.001 | 29.84 | 25.43 | Risk |
| GCS ≤ 8 | 190.52 | (160.22-226.55) | <0.001 | 380.54 | 319.95 | Risk |
| Hypothermic (< 36 C) | 2.71 | (2.27-3.23) | <0.001 | 4.86 | 3.96 | Risk |
| Other Mechanism | 2.06 | (1.66-2.57) | <0.001 | 3.54 | 2.70 | Risk |
| Penetrating Trauma | 2.80 | (2.25-3.50) | <0.001 | 5.05 | 3.92 | Risk |
| Private Vehicle | 0.25 | (0.18-0.35) | <0.001 | 7.38 | 5.12 | Protective |
| Air Transport | 1.94 | (1.75-2.15) | <0.001 | 3.29 | 2.89 | Risk |
| Other Transport | 2.49 | (1.56-3.96) | <0.001 | 4.41 | 2.50 | Risk |
| Helmet | 0.62 | (0.50-0.76) | <0.001 | 2.62 | 1.97 | Protective |

Note. E-values quantify the minimum strength of association that an unmeasured confounder would need to have with both the predictor and outcome, conditional on measured covariates, to explain away the observed association. aOR = adjusted odds ratio; CI = confidence interval; SBP = systolic blood pressure; mmHg = millimeters of mercury; $SpO_2$ = peripheral oxygen saturation; GCS = Glasgow Coma Scale; °C = degrees Celsius. Protective variables with aOR <1 were inverted before E-value calculation.

**Supplemental Table S3. Missing Data Assessment for Continuous Source Variables**

| Variable | Total N | N Missing (Overall) | % Missing (Overall) | N Missing (Ventilated) | % Missing (Ventilated) | N Missing (Not Ventilated) | % Missing (Not Ventilated) | SMD |
|---|---|---|---|---|---|---|---|---|
| Systolic Blood Pressure | 29,024 | 652 | 2.2% | 136 | 2.3% | 516 | 2.2% | 0.007 |
| Pulse Rate | 29,024 | 441 | 1.5% | 79 | 1.4% | 362 | 1.6% | -0.017 |
| Respiratory Rate | 29,024 | 834 | 2.9% | 306 | 5.2% | 528 | 2.3% | 0.157 |
| Pulse Oximetry (SpO2) | 29,024 | 1,061 | 3.7% | 214 | 3.7% | 847 | 3.7% | 0.001 |
| Glasgow Coma Scale | 29,024 | 899 | 3.1% | 166 | 2.8% | 733 | 3.2% | -0.018 |
| Temperature | 29,024 | 2,823 | 9.7% | 1,125 | 19.3% | 1,698 | 7.3% | 0.358 |
| Height | 29,024 | 5,028 | 17.3% | 878 | 15.1% | 4,150 | 17.9% | -0.077 |
| Weight | 29,024 | 582 | 2.0% | 134 | 2.3% | 448 | 1.9% | 0.025 |

Note. SMD = standardized mean difference; N = number of patients; $SpO_2$ = peripheral oxygen saturation. SMD reflects the standardized difference in missingness rates between ventilated and non-ventilated patients. An absolute SMD >0.10 was considered suggestive of meaningful differential missingness. The total analytic cohort included 29,024 patients.
